# Selection of Mother Wavelet Functions for Multi-Channel EEG Signal Analysis during a Working Memory Task

**DOI:** 10.3390/s151129015

**Published:** 2015-11-17

**Authors:** Noor Kamal Al-Qazzaz, Sawal Hamid Bin Mohd Ali, Siti Anom Ahmad, Mohd Shabiul Islam, Javier Escudero

**Affiliations:** 1 Department of Electrical, Electronic & Systems Engineering, Faculty of Engineering & Built Environment, Universiti Kebangsaan Malaysia, UKM Bangi Selangor 43600, Malaysia; E-Mail: sawal@eng.ukm.my; 2 Department of Biomedical Engineering, Al-Khwarizmi College of Engineering, Baghdad University, Baghdad 47146, Iraq; 3 Department of Electrical and Electronic Engineering, Faculty of Engineering, Universiti Putra Malaysia, UPM Serdang, Selangor 43400, Malaysia; E-Mail: sanom@upm.edu.my; 4 Institute for Microengineering and Nanoelectronics (IMEN), Universiti Kebangsaan, Malaysia (UKM), 43600 Bangi, Selangor, Malaysia; E-Mail: shabiul@ukm.edu.my; 5 Institute for Digital Communications; School of Engineering, University of Edinburgh, Edinburgh EH9 3JL, UK; E-Mail: Javier.escudero@ieee.org

**Keywords:** electroencephalography, memory, wavelet, multi-resolution analysis, cross-correlation

## Abstract

We performed a comparative study to select the efficient mother wavelet (MWT) basis functions that optimally represent the signal characteristics of the electrical activity of the human brain during a working memory (WM) task recorded through electro-encephalography (EEG). Nineteen EEG electrodes were placed on the scalp following the 10–20 system. These electrodes were then grouped into five recording regions corresponding to the scalp area of the cerebral cortex. Sixty-second WM task data were recorded from ten control subjects. Forty-five MWT basis functions from orthogonal families were investigated. These functions included Daubechies (db1–db20), Symlets (sym1–sym20), and Coiflets (coif1–coif5). Using ANOVA, we determined the MWT basis functions with the most significant differences in the ability of the five scalp regions to maximize their cross-correlation with the EEG signals. The best results were obtained using “sym9” across the five scalp regions. Therefore, the most compatible MWT with the EEG signals should be selected to achieve wavelet denoising, decomposition, reconstruction, and sub-band feature extraction. This study provides a reference of the selection of efficient MWT basis functions.

## 1. Introduction

Electroencephalogram (EEG) is a neurophysiological tool used to monitor and identify the changes in the brain signals associated with seizure disorder, traumatic brain injury, and other physiological problems [1]. EEG is also a widely available, cost-effective, and non-invasive tool. This tool can track *in vivo* brain functions in milliseconds with high temporal resolution by reflecting the inner mental tasks and pathological changes in the brain of a large population. EEG has also been utilized in cognitive science, neuropsychological research, clinical assessments, and consciousness research [2,3,4]. A typical clinical EEG frequency ranges from 0.01 Hz to approximately 100 Hz; the corresponding waveforms have an amplitude of a few microvolt to approximately 100 microvolt [5]. EEG background waveforms also convey valuable information; thus, these waveforms can be classified into five specific frequency power bands: delta band (δ), theta band (θ), alpha band (α), beta band (β), and gamma band (γ) [6,7]. In physiology, the extracted features from EEG signals provide a concise representation that shows the power distribution of an EEG signal in different frequency bands. Therefore, EEG power is the key to detecting interesting information related to cognitive and memory performance. Moreover, EEG power corresponds to the capacity of cortical information processing [8]. In this regard, two types of memory processes, namely, working memory (WM) and long-term memory, can be distinguished.

In our study, WM was considered. Based on an individual’s memory capacity, WM is the ability to maintain and manipulate information for brief periods. WM is considered as a temporary memory that can store approximately 7 ± 2 items for a short period (10–15 s up to 60 s) [9,10]. 

Several studies on EEG signal processing have been conducted to identify the brain activity patterns involved in cognitive process and memory [11,12,13,14]. For instance, Klimesch and others [8,15,16,17] have suggested that the changes in the cortical activity during WM tasks are related to the increase in δ, θ and γ magnitudes during memory load, whereas the α magnitude and the α/β power ratio decrease as WM load increases. 

EEG data are susceptible to contamination by artifacts that may introduce changes in the recorded cerebral activity. These artifacts may mimic brain cognitive or pathological activity; these artifacts may also overlap with EEG frequency bands with a larger amplitude than cortical signals. In general, several types of artifacts, including physiological and non-physiological artifacts, may corrupt the EEG data [18,19]. Physiological artifacts originate from generator sources in the body, such as heart, eye, and/or muscles, and cause cardiac, ocular, eye blinking, and muscular artifacts; by contrast, non-physiological artifacts, which are of technical origin, are related to environment and equipment [18,19]. 

Different techniques have been applied to overcome this problem because these artifacts directly affect EEG signal processing. Studies on artifact removal have also been proposed. For instance, He *et al.* [20] applied adaptive filtering to remove ocular artifacts. Romero *et al.* [21] proposed regression analysis, adaptive filtering, and independent component analysis (ICA) to reduce eye movement and obtained the best results through ICA [22]. Romero *et al.* [23] also used ICA to remove ocular artifact. Zeng *et al.* [24] performed empirical mode decomposition (EMD) as an adaptive method to detect and separate ocular artifacts from EEG signals. Li *et al.* [25] investigated the neuronal population oscillations using EMD Wavelet transform (WT) is a common and powerful denoising method widely applied to biomedical signals because of its localization characteristics of non-stationary signals in time and frequency domains [26,27,28]. WT has also been extensively utilized because this method can remove ocular artifact noise, eye blinking noise and cardiac artifacts [29,30,31,32,33]. Patel *et al.* [34] conducted a comparative study to remove ocular artifacts by using WT and EMD methods; WT with minimum signal distortion is more efficient than EMD [35]. Discrete wavelet transform (DWT) has also been considered as a promising technique to represent EEG signal characteristics by extracting features from the sub-band of EEG signals [28,36].

The selection of a mother wavelet (MWT) function is an important step and part of wavelet analysis to demonstrate the advantages of WT in denoising, component separation, coefficient reconstruction, and feature extraction from the signal in time and frequency domains. This step is necessary because studies have yet to provide specific MWT basis functions that cater to all EEG channels [28,36,37]. Several common standard families of WT basis functions, such as Haar (db1), Daubechies (db), Coiflets (coif), and Symlets (sym), are used. However, researchers have yet to establish well-defined rules for the selection of an efficient MWT basis function in a particular application or analysis. Despite the lack of such rules, a specific MWT becomes more suitable for a specific application and signal type because of WT properties. The selection of MWT can be either empirical or dependent on the visual inspection of the repeated signal pattern accompanied by previous experiences and knowledge [38]. Adeli *et al.* and several researchers have investigated Daubechies family of different orders “db2”, “db3”, “db4”, “db5”, and “db6”, particularly “db4”, exhibits the highest cross-correlation with epileptic spike signals [28,39,40,41]; “db2” is more appropriate EEG smoothing [42]. Zikov *et al.* [43] chose “coif3” because its shape resembles that of eye blink artifacts. Andrade *et al.* [44] used “db5” to remove noise from EMG signals; Andrade *et al.* [45,46] also utilized “db4”, “sym7”, “coif3”, “coif4”, and “coif5” to enhance ECG detection. However, a more precise selection of a MWT basis function remains a challenge because the properties of the WT functions and the characteristic of the signal to be analyzed should be carefully matched.

Considering these findings, we conducted a comparative study to select the best MWT basis function of the characteristics of EEG datasets in a WM task. Forty-five MWTs, including Daubechies (db1–db20), Symlets (sym1–sym20), and Coiflets (coif1–coif5), were used to evaluate their compatibility with the EEG dataset. The similarities of these MWT functions to be matched to the recorded EEG dataset were also analyzed using a cross-correlation method (XCorr). Furthermore, significant differences in the selected MWT base functions among the scalp regions were evaluated through one-way ANOVA. The selection of optimal MWT is useful in denoising, decomposition, significant component reconstruction, and feature extraction from the EEG signal sub-bands that were used to understand the brain functions and reveal the hidden characteristics in the EEG spectra.

## 2. Methods

**Figure 1:**
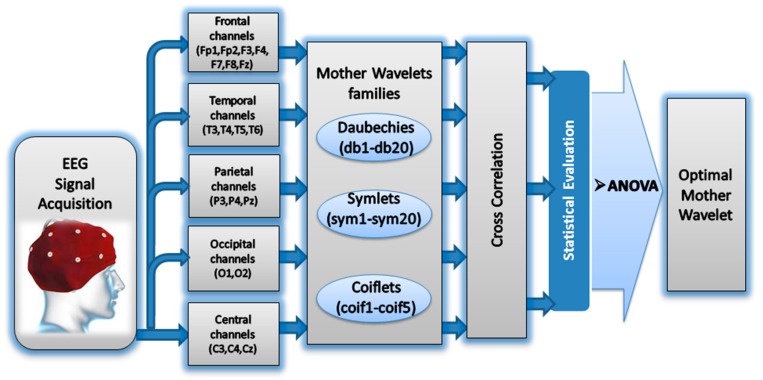
The block diagram of the proposed method.

Figure 1 shows the general block diagram of our proposed approach for selecting the optimal mother wavelet function among 45 functions.

The EEG dataset was originally acquired from 19 sites on the scalp by using cap electrodes. Conventional filtering methods were used as an initial stage to process the 19 channels of the EEG data. A notch filter at 50 Hz was used to remove the power line interference noise; a band pass infinite impulse response filter with a frequency range of 0.5 Hz to 64 Hz was used to limit the band of the recorded EEG signals.

### 2.1. Subjects and EEG Recording Procedure

Ten EEG recordings were examined in this study. These EEG datasets were recorded from ten healthy control subjects composed of six males and four females aged 47.9 ± 6.5 years (mean ± standard deviation, SD). The subjects were recruited from the Pusat Perubatan Universiti Kebangsaan Malaysia, the Medical Center of the National University of Malaysia. A critical concept in enrolment of the volunteers was that the subjects did not have a previous history of mental and neurological abnormalities. The neuropsychological assessments have been used to assess the volunteers and identifying the normal reference control subjects in order to enroll them. These subjects also underwent cognitive evaluation, including mini-mental state examination (MMSE) [47] and Montreal Cognitive Assessment (MoCA) [48] which involves tests of a variety of cognitive domains abilities including attention, memory, language, and orientation. The results of the working memory test performance were included within the MMSE and MoCA (attention and concentration parts of these assessments). All the control subjects were getting the maximum score in working memory test performance part to be included in our study. Besides, all the control subjects remembered (enumerated all the five words at the enumerate words step) at the end of EEG recording. Table 1 shows the socio-demographic and neuropsychological data of the control healthy subjects.

**Table 1:** Sociodemographic data of the control subjects. Mini-Mental State Examination (MMSE) and Montreal Cognitive Assessment (MoCA) scores are also shown, (Age in years, MMSE and MoCA, mean ± standard deviation SD).

Demographic and Clinical Features	Control
Number	10
Age	47.9 ± 6.5
MMSE	29.7 ± 0.67
MoCA	28.9 ± 0.87
Female/Male	4F/6M

**Figure 2:**
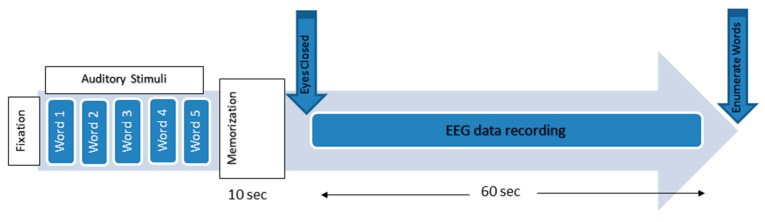
The working memory experimental paradigm.

**Figure 3:**
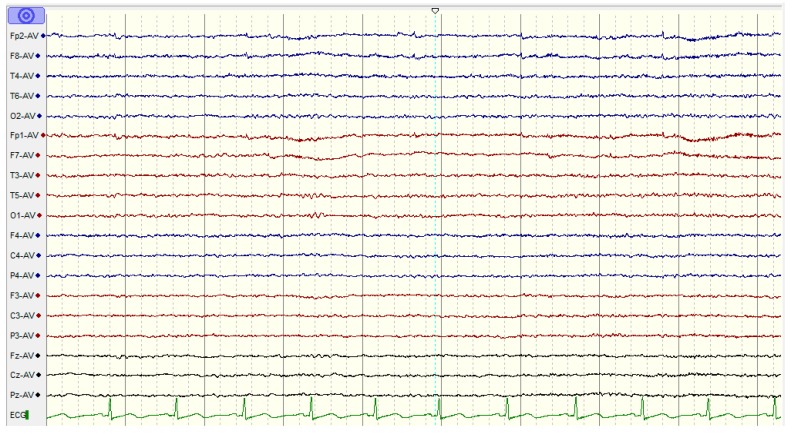
The EEG activities for a healthy subject during a working memory task using the NicoletOne systems (V32).

The experimental protocols were approved by the Human Ethics Committee of the National University of Malaysia. Information consent forms were signed by the participants. The EEG activities were recorded using a NicoletOne system (V32), which was designed and manufactured by VIASYS Healthcare Inc. (Middleton, WI 53562, USA). A total of 19 electrodes, plus the ground and system reference electrodes, were positioned on the basis of the 10-20 international system (Fp1, Fp2, F7, F3, Fz, F4, F8, T3, T5, T4, T6, P3, Pz, P4, C3, Cz, C4, O1, and O2). The NicoletOne EEG system was sampled using referential montage at a sampling frequency of 256 Hz, an impedance of electrode/skin of <10 kΩ, sensitivity of 100 µV/cm, a low cut of 0.5 Hz, and a high cut of 70 Hz. The subjects were asked to be motionless as possible and to memorize five words for 10 s. Subsequently, they were asked to remember the five words while the EEG data were recorded with their eyes closed. They were then instructed to open their eyes after 1 min and enumerate all they could remember from the five words (Figure 2) [49]. EEG data were recorded for 60 s during the WM task (Figure 3).

### 2.2. Wavelet Analysis

WT is a powerful spectral estimation technique for the time–frequency analysis of a signal. WT is an effective denoising method introduced to address the problem of non-stationary signals, such as EEG, electrocardiography (ECG), electromyography (EMG), and ocular artifacts [29,30,31,50]. The multi-resolution analysis (MRA) method provides varying time–frequency resolutions in all frequency ranges. MRA provides varying resolution at different time and frequency [51,52]. It is designed to provide a good time resolution and a poor frequency resolution at high frequency and a good frequency resolution and a poor time resolution at low frequency. Moreover, WT can be applied to solve resolution-related problems by dividing the data of interest into different frequency components and by evaluating each component with a resolution matched to its scale [53,54]. Discrete wavelet transform (DWT), which has less computational time than continuous WT, is a fast and non-redundant transform used to analyze low- and high-frequency components in the EEG signals [55]. DWT can be processed by obtaining the discrete value of the parameters a and b, as in Equation (1). DWT can be obtained as a set of decomposition functions of the correlation between the signal f(t) and the shifting and dilating of one specific function called the MWT function ψ(t). MWT is shifted by the location parameter (b) and dilated or contracted by the frequency scaling parameter a, as in Equation (2) [52,56,57,58]:

(1) DWTm,n(f)=a0−m2∫​f(t)ψ(a0−mt−nb0)dt

$a_{0}$ and $b_{0}$ values are set to 2 and 1, respectively, R are the real numbers:

(2) $$\psi_{a,b}\left(t\right)=\frac{1}{\sqrt{a}}\psi \left(\frac{t-b}{a}\right),\;a\epsilon R^{+},\;b\in R$$

Mallat [54] developed a method by which DWT is implemented; in this method, the DWT decomposes a signal into different frequency bands by passing it through two quadrature mirror filters via a finite impulse response, where g is a high-pass filter (HPF) and h is a low-pass filter (LPF). h is related to the scaling function, whereas g is related to the MWT, as in Equations (3)–(5) [54]:

(3) $$g\left(h\right)={\left(-1\right)}^{n}h\left(1-n\right)$$

(4) $$\phi \left(x\right)=\sum_{n}h\left(n\right)\sqrt{2}\phi \left(2x-n\right)$$

(5) $$\psi \left(x\right)=\sum_{n}g\left(n\right)\sqrt{2}\;\phi \left(2x-n\right)$$

The QMF output is characterized as shown in Equations (6) and (7):

(6) $$H_{L}=\sum_{n}h\left(n-2L\right)x\left(n\right)$$

(7) $$G_{L}=\sum_{n}g\left(n-2L\right)x\left(n\right)$$

The signal x(n)  convolves with h(n−2L) when this signal acts as an LPF; otherwise, this signal acts as an HPF and convolves with g(n−2L). The result transforms the original signal into two sub-bands [0−FN2] and [FN2−FN]. $H_{L}$ is the approximation component A that represents low-resolution components; $G_{L}$ is the detail decomposition component D that describes high-resolution components [59,60]. Several parameters, including the selected MWT, wavelet decomposition level, and selected threshold, should be selected carefully when WT-based processing methods are used.

#### 2.2.1. Mother Wavelet Optimal Selection

In most cases, optimal MWT functions are selected on the basis of the compatibility with the EEG signal characteristics to be analyzed. Accurate MWT selection not only helps retain the original cortical signal but also enhances the frequency spectrum of the denoised signal [61]. However, several common standard wavelet families, including Daubechies, Symlets, Coiflets, Morlet, Mexicanhat, and Meyer wavelets, are considered [55]. A critical point in EEG signal processing via WT is the selection of a suitable MWT and decomposition level to reduce the artifacts that contaminate EEG signals. The selection of the base WT function from the WT families also depends on their characterization of orthogonality [62]. Therefore, the use of WT basis function from orthogonal families, such as Daubechies, Coiflets, and Symlets, helps conserve the decomposed EEG signal and obtain optimal reconstructed signals [63]. These MWTs are regarded as the most common parameters in biomedical signal processing [50,64,65,66].

**Figure 4:**
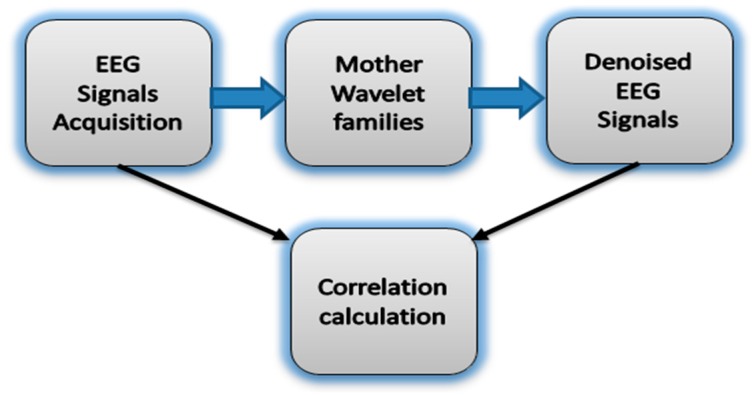
The block diagram of the correlation between the noisy EEG signals and denoised EEG signals using mother wavelet families.

To reduce computational complexity and to ensure an effective denoising procedure of the EEG signal to unique reconstructed signal, we selected 45 MWTs from three different orthogonal families, including Daubechies (db1–db20), Symlets (sym1–sym20), and Coiflets (coif1–coif5) [64,65,67,68]. These MWTs share orthogonality properties necessary to extract high- and low-frequency details from the original signal without losing information. The correlation XCorr between the band-limited EEG signals of interest X and the wavelet denoised signal Y (Figure 4) is expressed in Equation (8) [65,69]:

(8) $$XCorr\left(X,Y\right)=\frac{\sum^{\text{​}}\left(X-\bar{X}\right)\left(Y-\bar{Y}\right)}{\sqrt{\sum^{\text{​}}{\left(X-\bar{X}\right)}^{2}{\left(Y-\bar{Y}\right)}^{2}}}$$

where $\bar{X}$ and $\bar{Y}$ are the mean value of the X and Y, respectively.

**Figure 5:**
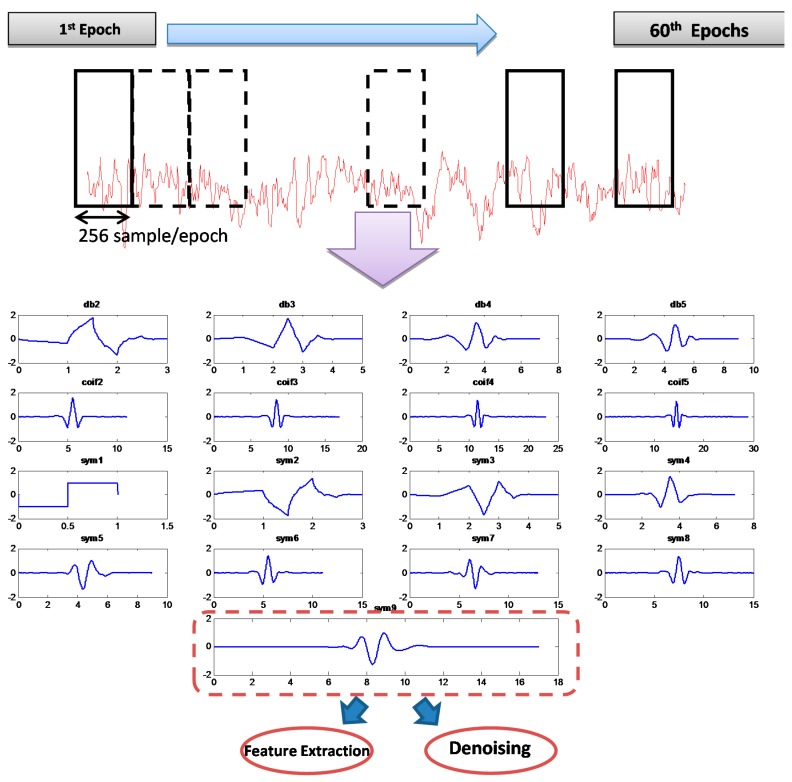
Noisy EEG epochs and mother wavelet of Daubechies (db order from 2 to 5), Coiflets (coif order from 2 to 5) and symlet (sym order from 1 to 9) representation.

MWT was chosen by dividing each of the recorded 19 channels with a total length of 15,360 samples into 60 epochs; the length of each epoch was 256 data points (one segment), as shown in Figure 5. All of the MWTs were used to verify the correlation of the MWT basis function with a specific segment.

The XCorr results of the 19 channels were grouped into five recording regions corresponding to the scalp region. These regions include the frontal region of the seven frontal channels (Fp1, Fp2, F3, F4, F7, F8, and Fz), the temporal region of the four temporal channels (T3, T4, T5, and T6), the parietal region of the three parietal channels (P3, P4, and Pz), the occipital region of the two occipital channels (O1 and O2), and the central region of the three central channels (C3, C4, and Cz).

#### 2.2.2. Level of Decomposition and Threshold Selection

The selection of a suitable number of decomposition levels is necessary to analyze in EEG signal analysis using DWT. Therefore, the number of decomposition levels can be chosen on the basis of the dominant frequency of signals and the usefulness of the extracted features from individual wavelet components [70,71,72,73]. Once the wavelet basis function and the decomposition level are specified, MRA methods can be performed.

In this study, the sampling frequency was 256 Hz, the band-limited EEG was then subjected to a five-level decomposition coefficient of six sub-band signals through DWT. The six sub-bands, particularly cD1, cD2, cD3, cD4, cD5 and cA5, represented the frequency range from the band-limited EEG signal, where cA is the decomposition approximation coefficient and cDs are the decomposition detail coefficients. Threshold limit and function are relevant factors to extract meaningful information by employing the WT denoising technique. Considering this finding, [74,75,76,77] proposed a WT threshold value by calculating the noise level of all WT sample coefficients, and then setting the threshold values to reveal noise-free WT coefficients. The SURE threshold, is an adaptive soft thresholding method, which is finding the threshold limit for each level based on Stein’s unbiased risk estimation [78] and commonly used value in [79,80,81].

**Figure 6:**
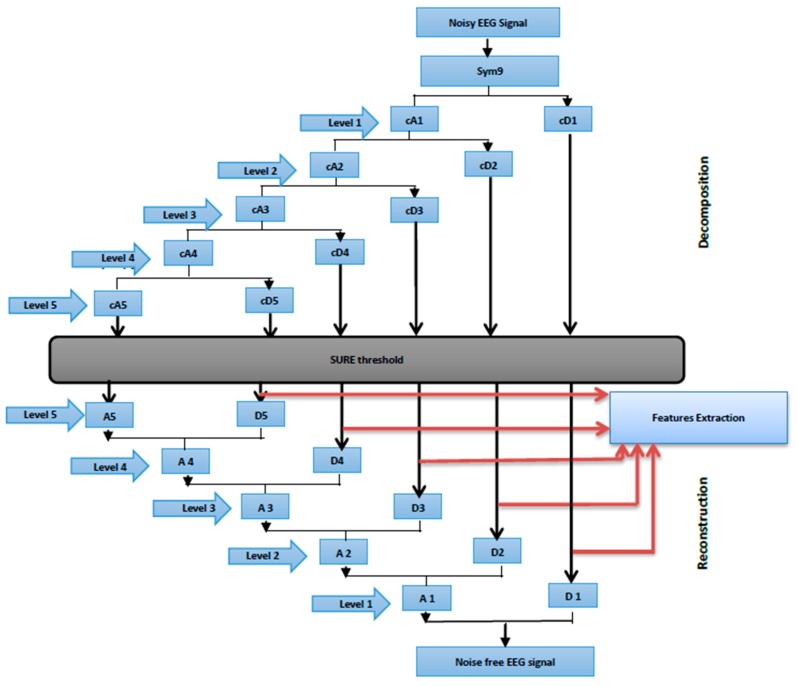
Wavelet Multi-resolution analysis.

Once the threshold coefficients were extracted from each level, the effect of the noises on the EEG signals were removed. The signals at each level were reconstructed using inverse DWT (Figure 6). The first reconstructed details D1 is considered to be mainly the noise components of the EEG signal (such as muscular artifacts), the four reconstruction details of the sub-band signals D2–D5 and the reconstruction approximation of the sub-band signal A5 yielded signal information related to each EEG frequency band (see Table 2).

**Table 2:** The EEG signal decomposition into five frequency bands and noise.

Decomposition Levels	Frequency Bands (Hz)	Decomposed Signals	EEG Bands
**1**	64–128	D1	Higher gamma and noise
**2**	32–64	D2	Lower gamma (γ)
**3**	16–32	D3	Beta (β)
**4**	8–16	D4	Alpha (α)
**5**	4–8	D5	Theta (θ)
**5**	0–4	A5	Delta (δ)

The sub-band features of the extracted wavelet coefficients provided a concise representation of the band-limited EEG signal. In addition, WT uses a variable window size across the whole signal length that helped quantify the changes in EEG in different frequency bands. In this research, the relative power (*RP*) in alpha (α*RP*), beta (β*RP*), theta (θ*RP*), delta (δ*RP*), and gamma (γ*RP*) were calculated to obtain the WT decomposed signals and to specify the changes in *RP* during the WM task. The *RP* of each of the selected frequency bands α, β, θ, δ, and γ can be calculated using Equation (9) [82]:

(9) $$RP\left(\text{\%}\right)=\frac{\sum^{\text{​}}\text{Selected frequency range }}{\sum^{\text{​}}\text{Total range }\left(0.5-64\text{ Hz}\right)}$$

### 2.3. Statistical Analysis

Normality was assessed through Kolmogrov–Smirnov test; homoscedasticity was verified with Levene’s test. Statistical analysis were performed through ANOVAs in SPSS 22. In the first session of ANOVA, the significant differences among the five groups of the scalp regions and the 45 MWTs were evaluated using *XCorr* as the dependent variable. A second session of ANOVA was performed on the *RP*. The significant differences among the five groups of the scalp regions and *RP* as dependent variable were evaluated. Post-hoc comparison was performed through Duncan’s test. The significance was set at *p* ˂ 0.05.

## 3. Results and Discussion

**Figure 7:**
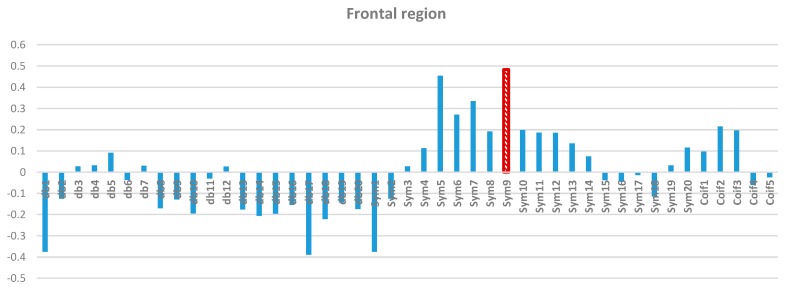
Comparative plot of correlation coefficients with 45 mother wavelet filter for the frontal region of the brain for 10 control subjects.

**Figure 8:**
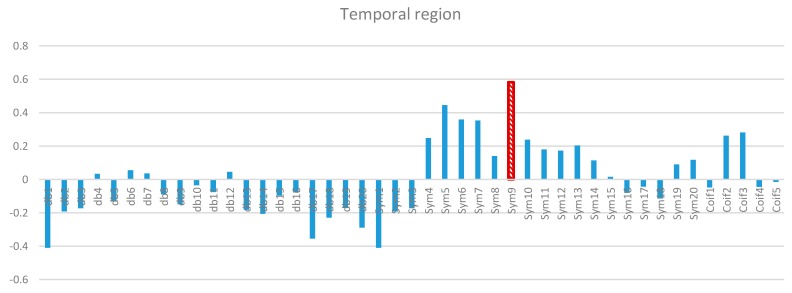
Comparative plot of correlation coefficients with 45 mother wavelet filter for the temporal region of the brain for 10 control subjects.

**Figure 9:**
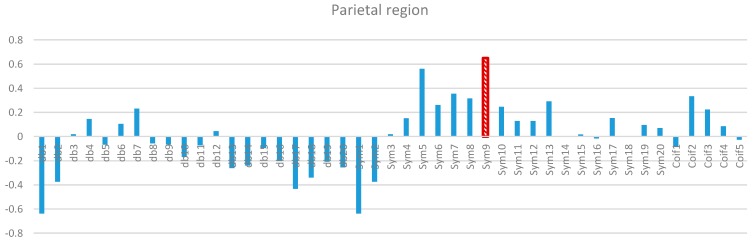
Comparative plot of correlation coefficients with 45 mother wavelet filter for the parietal region of the brain for 10 control subjects.

**Figure 10:**
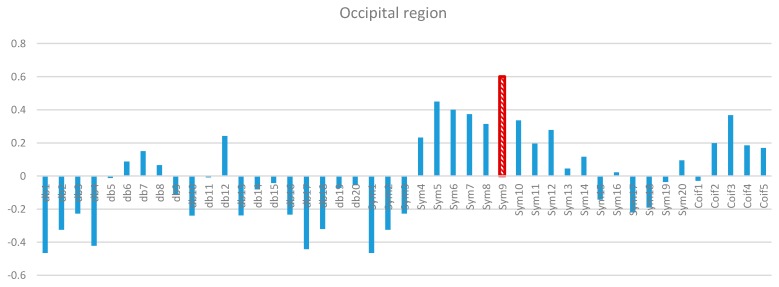
Comparative plot of correlation coefficients with 45 mother wavelet filter for the occipital region of the brain for 10 control subjects.

**Figure 11:**
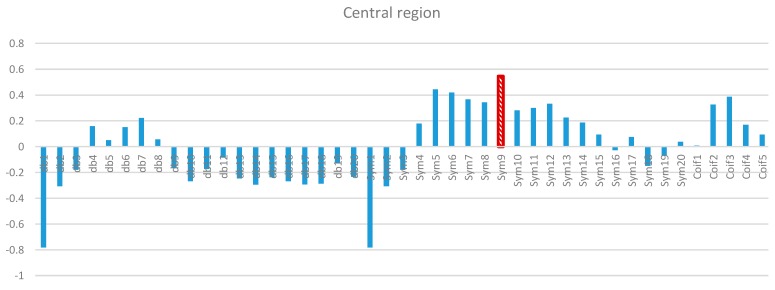
Comparative plot of correlation coefficients with 45 mother wavelet filter for the central region of the brain for 10 control subjects.

Two-way ANOVA was performed before the data were analyzed to determine the best MWT of the frontal, temporal, parietal, occipital, and central regions of the scalp. The results of normality test revealed that the dependent variable XCorr was distributed normally in all of the regions. The results of homogeneity test showed that the variances among groups were homogeneous. The results of ANOVA demonstrated that the 45 MWTs significantly differed. A *post-hoc* test using the Duncan multiple ray test showed that the highest mean in the frontal region (Fp1, Fp2, F3, F4, F7, F8, and Fz) channels belonged to “sym9”, which was significantly different from all of the MWTs except “sym5”, and “sym7” (Figure 7). In the temporal region (T3, T4, T5, and T6) channels, the highest mean that significantly differed from all of the MWTs belonged to “sym9”, which was significantly different from all of the MWTs except “sym5” (Figure 8). The parietal region (P3, P4, and Pz) channels shared the temporal region and obtained the same results; the highest mean belonged to “sym9”, which was significantly different from all of the MWTs except “sym5” (Figure 9). Furthermore, the occipital region (O1 and O2) channels, the highest mean belonged to “sym9” (Figure 10). The central region (C3, C4, and Cz) channels, shared the occipital region and obtained the same results; “sym9” was significantly different from all of the MWTs (Figure 11).

**Figure 12:**
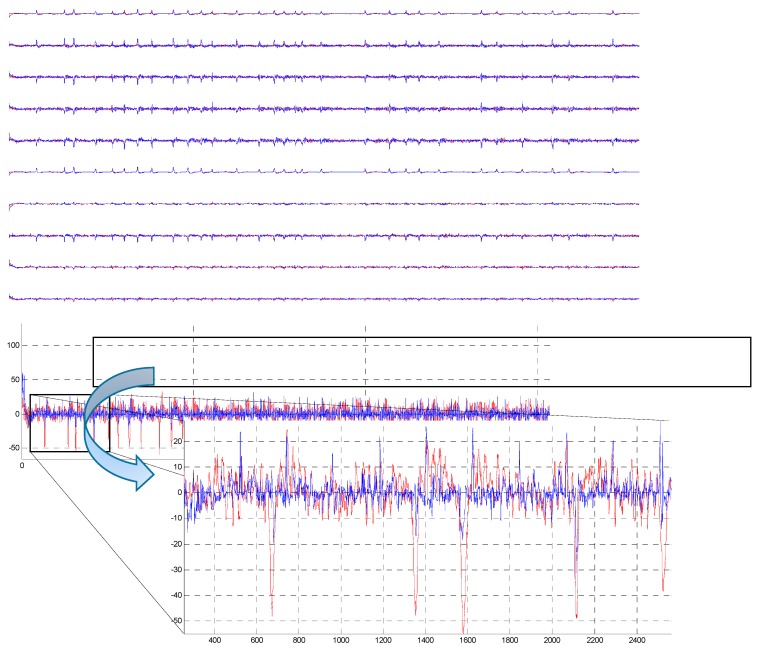
The removal results after the “sym9” MWT were applied on the EEG channels, the EEG signals before artifact removal (in red), the EEG signals after denoising (in blue).

Wavelet denoising method using “sym9” has been applied to each individual channel of the EEG dataset (Figure 12). It can be observed that the ocular artifacts are sufficiently removed (the blue), in contrast to the original recorded EEG (the red). 

**Figure 13:**
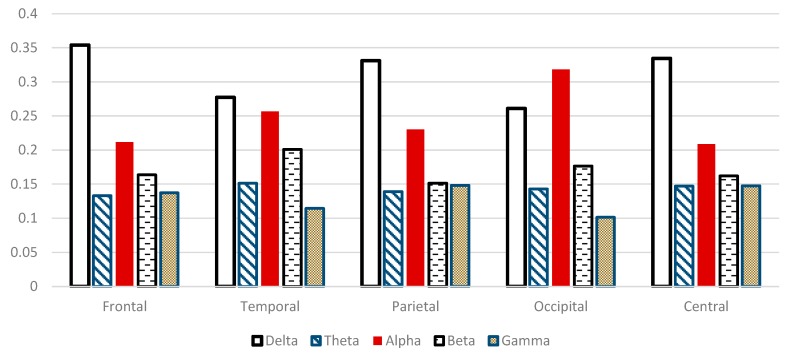
Comparative plot of the relative powers after using “sym9” wavelet filter for the five scalp regions of the brain for 10 control subjects.

Figure 13 shows the relative spectral power changes in the five scalp regions during WM tasks examined with EEG. δ*RP* is significantly higher in frontal, central, parietal and temporal regions (*P* < 0.05). Moreover, θ*RP* is significantly higher in temporal, central, occipital and parietal regions (*P* < 0.05). Furthermore, γ*RP* is significantly higher in central, parietal and frontal regions (*P* < 0.05) during WM task. On the contrary, α*RP* components are significantly lower in central and frontal region compared to other scalp regions. Interestingly, α*RP* has had the highest component in the occipital region, this may be related to eyes closing during WM task. β*RP* components are significantly smaller in parietal, frontal and central regions, but higher components in the temporal and occipital regions (*P* < 0.05). Our findings regarding the spectral analysis agreed other studies. For instance, Klimesch described the changes in the brain activity which are strongly associated with cognitive and attentional working memory performance as decreasing in both alpha and beta but increasing in both delta and theta in [8]. Gevins *et al.* attributed the changes during working memory task to alpha and theta. Frontal central theta increased due to memory load whereas decreasing in central alpha during working memory load [15]. Finally, Lundqvist *et al.* correlated the changes in brain activity to encoding one or more items in WM and these changes have associated with increase in theta and gamma and decrease in alpha and beta power [16].

This study showed several limitations. For instance, the sample size was small and an additional analysis with a large database should be performed in the future. Add to that a potential limitation for high gamma bands which were not used in this analysis, due to the cut off frequency of the EEG recording system used in this study, potentially missing information there but that such spectral range is particularly noisy as well due to muscular artifacts. Moreover, one MWT function cannot be applicable to all physiological and pathological states of the brain. Besides, previous studies focused on the selection of a MWT compatible with ECG and epileptic seizures [67,68]. For instance, Rafiee *et al.* [65] used two multi-channel datasets for EMG signals and three-channel datasets for EEG signals to select the most suitable MWT function for human biological signals; Kang *et al.* [83] proposed two-channel EEG electrode on the frontal region of the scalp to evaluate the frontal region simulated by mental workload. Singh *et al.* [67] examined a single-bit ECG signal. Messer *et al.* [55] used single phonocardiogram (PCG) signal to select the best MWT to remove the noise from PCG. Despite these drawbacks, this study may provide a method to identify suitable MWT functions for each region of the scalp during a WM task. The selection of optimal MWTs is necessary to achieve the most efficient denoising, decomposition, reconstruction, and feature extraction. In this manner, valuable physiological information can be retained to help improve diagnostic procedures through the adroit integration of wavelet denoising and sub-band feature extraction. EEGs are commonly used to diagnose epilepsy [36,84] and other neurological disorders, such as Alzheimer’s disease [85,86,87,88], and attention deficit hyperactivity disorder (ADHD) [89]. 

## 4. Conclusions

Different types of artifacts contaminate EEG signals. In this study, the compatibility of 45 MWT basis functions from the Daubechies, Symlets, and Coiflets orthogonal families were selected and subjected to analysis because of their similarities in the five scalp regions (the 19 EEG channels) during the WM task. We successfully selected an optimal wavelet function with the best performance for denoising and the highest compatibility with the EEG datasets of the ten control subjects. However, the selection of MWT functions was based on the best XCorr results between the recorded EEG signals and the WT denoising results. 

**Figure 14:**
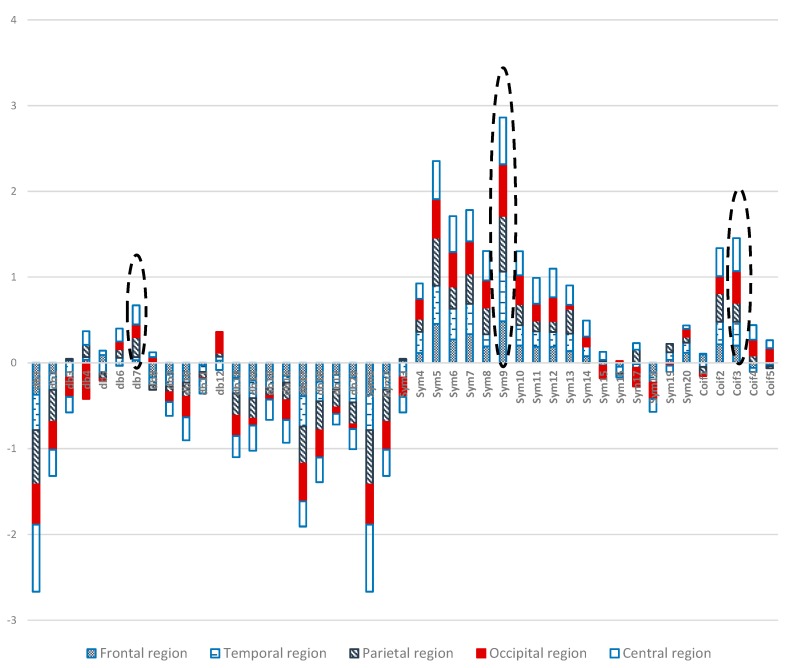
Comparative plot of the correlation coefficients with 45 mother wavelet filter for the 5 regions of the brain for 10 control subjects.

On the basis of Figure 14, we can conclude that “sym9” from the Symlets family exhibits the highest similarities and compatibilities with the recorded EEG signals in all of the five scalp regions. Remarkable results were demonstrated by “coif3” and “db7” from the Coiflet and Daubechies families, respectively. Indeed, these results may be attributed to the similarity between “sym9” and the EEG signal recorded from the scalp regions during the WM task; “coif3” and “db7” may resemble the EEG signals that appeared in the regions during memory load. Therefore, the most compatible MWT with the 19 EEG channels should be selected to perform wavelet denoising and decomposition. The selection method can also be considered as a complementary tool to help physicians diagnose diseases by using EEG data.

In the future, our aim to analyze the EEG background activity in dementia patients starting from EEG signal acquisition, followed by EEG signal preprocessing stages using wavelet denoising method for signal enhancement, linear and non-linear features extraction will be the next focus to cater for the fluctuations of EEG signal and end with classification methods to discriminate dementia degree of severity.
